# RUNX3 Suppresses Migration, Invasion and Angiogenesis of Human Renal Cell Carcinoma

**DOI:** 10.1371/journal.pone.0056241

**Published:** 2013-02-14

**Authors:** Feifei Chen, Jin Bai, Wang Li, Pengjin Mei, Hui Liu, Linlin Li, Zhenqiang Pan, Yongping Wu, Junnian Zheng

**Affiliations:** 1 Jiangsu Key Laboratory of Biological Cancer Therapy, Xuzhou Medical College, Xuzhou, Jiangsu, China; 2 The Affiliated Hospital of Xuzhou Medical College, Xuzhou, Jiangsu, China; 3 School of Pathology, Xuzhou Medical College, Xuzhou, Jiangsu, China; 4 Department of Oncological Sciences, Mount Sinai School of Medicine, New York, New York, United States of America; University of Alabama at Birmingham, United States of America

## Abstract

RUNX3 (runt-related transcription factor-3) is a known tumor suppressor gene which exhibits potent antitumor activity in several carcinomas. However, little is known about the role of RUNX3 in human renal cell carcinoma (RCC). To investigate the clinical relevance of RUNX3 in RCC patients, immunohistochemistry was performed to detect the clinical relevance of RUNX3 in 75 RCC tissues and paired non-cancerous tissues by using tissue microarray (TMA). We also investigated the role of RUNX3 in RCC cell migration, invasion and angiogenesis. The RUNX3 expression was decreased dramatically in human RCC tissue. The RUNX3 expression was significantly correlated with tumor size (*P*<0.001), depth of invasion (*P*<0.001), and of TNM stage (*P*<0.001). Restoration of RUNX3 significantly decreased renal carcinoma cell migration and invasion capacity compared with controls. In addition, we found that overexpression of RUNX3 reduced the proliferation and tube formation of human umbilical vascular endothelial cells (HUVECs). Gelatin zymography and Western blot showed that RUNX3 expression suppressed matrix metalloproteinase-9 (MMP-9) protein level and enzyme activity. Western blot and ELISA showed that RUNX3 restoration inhibited the expression and secretion of vascular endothelial growth factor (VEGF). Taken together, our studies indicate that decreased expression of RUNX3 in human RCC tissue is significantly correlated with RCC progression. Restoration of RUNX3 expression significantly inhibits RCC cells migration, invasion and angiogenesis. These findings provide new insights into the significance of RUNX3 in migration, invasion and angiogenesis of RCC.

## Introduction

RCC is the most common carcinoma of the adult kidney, accounting for the majority (90%) of kidney cancer cases. Its incidence has gradually increased during the last decades [1]. At present, surgical resection is the most effective treatment for localized RCC tumors. However, 30% of patients develop metastatic disease after surgery [2], and median survival of those patients is only about 13 months [3]. Therefore, novel diagnostic and therapeutic markers are urgently needed for this disease. Discovery of biomarkers and their application in conjunction with traditional cancer diagnosis, clinical staging, and prognosis would contribute to improving early diagnosis and patient therapy.

The RUNX family members, RUNX1, RUNX2 and RUNX3, encode DNA-binding α subunits that bind a common β subunit, CBFβ, to generate heterodimeric transcription regulators [4]. All three RUNX family members play important roles in normal developmental processes and carcinogenesis [5]. Among the three RUNX family members, RUNX3, in particular, has been shown to play a tumor suppressor role in several cancers and its expression levels are down-regulated in cancer tissues [6], [7]. Analysis of clinical tissue samples from peritoneal metastases arising from gastric cancers showed that RUNX3 expression decreased significantly in the metastatic tissue, compared to normal gastric mucosa or primary main tumors [8]. Importantly, the decrease in RUNX3 protein expression is significantly associated with decreased survival of gastric cancer and melanoma patients [9], [10]. These studies suggest a meaningful role for RUNX3 in the tumorigenesis of human cancers. There has been evidence that RUNX3 can function as a tumor suppressor by regulating cancer growth and angiogenesis [11]. In our previous study, we demonstrated that RUNX restoration suppressed glioma cell migration and invasion ability [12]. However, less is known about the expression and function of RUNX3 in RCC.

In the present study, we evaluated RUNX3 staining in 75 RCC tissues and paired non-cancerous tissues using tissue microarray technology a immunohistochemistry and analyzed the correlation between RUNX3 expression and clinicopathologic variables. Our data demonstrated that decreased expression of RUNX3 was significantly associated with RCC progression. In addition, we found that restoration of RUNX3 expression in human renal cancer cells dramatically decreased cell migration and invasion abilities by down-regulating MMP-9 expression. We also found that overexpression of RUNX3 markly suppressed angiogenesis, which correlated with down-regulation of VEGF. The data indicate that RUNX3 may be a tumor suppressor involved in the progression of RCC.

## Materials and Methods

### Ethics Statement

This study was performed under a protocol approved by the Institutional Review Boards of Affiliated Hospital of Xuzhou Medical College and all examinations were performed after obtaining written informed consents.

### Patients and samples

A RCC tissue microarray (TMA) was purchased from Shanghai Xinchao Biotechnology (Shanghai, China). Pathologic grades of tumors were defined according to the WHO criteria as follows: seventy-five cases of RCC tissues and paired non-cancerous tissues (Grade I, II, III and IV). The array dot diameter was 1.5 mm, and each dot represented a tissue spot from one individual specimen that was selected and pathologically confirmed. Three RCC tissues and paired non-cancerous tissues were obtained from the affiliated hospital of Xuzhou Medical College.

### Immunohistochemistry of TMA

Immunohistochemistry was performed according to the streptavidin-peroxidase (Sp) method using a standard Sp Kit (Zhongshan biotech, Beijing, China). The TMA slide was incubated with monoclonal mouse anti-RUNX3 antibody (1∶200) (Medical and Biological Laboratories, Nagoya, Japan) overnight at 4°C, and diaminobenzidine (DAB; Zhongshan Biotech, Beijing, China) was used to produce a brown precipitate. The immunoreactivity was assessed blindly by two independent observers using light microscopy (Olympus BX-51 light microscope), and the image was collected by Camedia Master C-3040 digital camera. The expression of RUNX3 was graded as positive when 5% of tumor cells showed immunopositivity. Biopsies with <5% tumor cells showing immunostaining were considered negative [9].

### Cell lines and transfection

Human RCC cell lines 786O and ACHN were purchased from the Shanghai Institute of Biochemistry and Cell Biology, Chinese Academy of Sciences (Shanghai, China). Human umbilical vascular endothelial cells (HUVECs) were obtained from KeyGEN biotech (Nanjing, China). 786O cells were cultured in RPMI1640 medium supplemented with 10% fetal calf serum (Invitrogen, Shanghai, China), ACHN cells and HUVECs were cultured in DMEM medium supplemented with 10% fetal calf serum. Cells were in a 37°C humidified incubator with 95% air, 5% CO_2_.

The pFlag-control and pFlag-RUNX3 expression plasmids were obtained from Dr Pei-Jung Lu (National Cheng-Kung University, Tainan, Taiwan). Transfection of the pFlag-control and the pFlag-RUNX3 plasmids into the renal carcinoma cells were carried out using Lipofectamine 2000 transfection reagent (Invitrogen, Shanghai, China) following the manufacturer's protocol.

### Migration assay

Cell migration was determined by using a modified two chamber migration assay with a pore size of 8 µm. For migration assay, 1×10^5^ 786O and ACHN cells were seeded in serum-free medium in the upper chamber. After 12 h incubation at 37°C, cells in the upper chamber were carefully removed with a cotton swab and the cells that had traversed the membrane were fixed in methanol and stained with leucocrystal violet. The number of invasive cells was determined by counting the leucocrystal violet-stained cells. For quantification, cells were counted under a microscope in five fields (up, down, median, left, right. ×200).

### Invasion assay

The invasion assay was performed using a modified two chamber plates with a pore size of 8 µm. The transwell filter inserts were coated with matrigel (BD Biosciences, NJ, USA). 0.5×10^5^ 786O cells and 1×10^5^ ACHN cells were seeded in serum-free medium in the upper chamber. After 24 h incubation at 37°C, noinvasive cells were gently removed from the top of the matrigel with a cotton-tipped swab. Invasive cells at the bottom of the matrigel were fixed in methanol, stained with leucocrystal violet and counted.

### Cell proliferation assay

Cellular proliferation was assayed using Cell counting kit-8 (CCK-8) purchased from Beyotime Institute of Biotechnology (Nanjing, China). In brief, 2×10^4^ HUVECs suspended in 100 µl conditioned medium from either control cells or RUNX3 transfected cells. HUVECs were seeded at a density of 2×10^4^ in a 96-well culture plate and incubated at 37°C in a humidified atmosphere containing 5% CO_2_ for 24 h. Then, cell proliferation was detected according to the manufacturer's instructions.

### Endothelial cell tube formation assay

Transfected 786O and ACHN cells (1×10^6^) were cultured in 6-well plate with fresh complete medium for 24 h, and the medium was collected and centrifuged to remove any cell debris before its use as a conditioned medium. 48-well plate was coated with Matrigel and kept in 37°C for 30 min. Then, 2×10^4^ HUVECs were suspended in 100 µl conditioned medium and applied to the pre-coated 48-well plate. After incubation at 37°C for another 24 h, the number of capillary-like tubes from three randomly chosen fields was counted.

### Western blot analysis

For patients' tissues, the homogenates were centrifuged at 1,000 g for 10 min at 4°C and the supernatants were collected. For cancer cells, twenty-four hours after transfection, cells were harvested from the plates. Patients' tissue protein and aliquots of cell extracts were separated on a 12% SDS-polyacrylamide gel. The proteins were then transferred to nitrocellulose membrane and incubated overnight at 4°C with the following antibodies: mouse anti-RUNX3 (Medical and Biological Laboratories, Nagoya, Japan), rabbit anti-MMP-9 (Cell Signaling Technology, Beverly, MA, USA), mouse anti-VEGF (Santa Cruze, CA, USA) and mouse anti-β-actin (Boster Biotechnology, Wuhan, China). Membranes were then washed and incubated with secondary antibody (goat anti-rabbit and goat anti-mouse IgG) for 2 h, stained by coloration fluid which contains 10 ml alkaline phosphatase buffer, 33 µl BCIP, and 66 µl NBT, and finally, the membrane is scanned. Each blot was repeated three times.

### Gelatin zymography

2×10^6^ cells were seeded in 100-mm plate for 24 h. The proteins in the conditioned medium were concentrated with Ultracel-30 k centrifugal filters (Millipore, Billerica, USA) at 5,000 g for 15 min at 4°C. Twenty-five microgram of the proteins was loaded in non-redenaturing conditions on a 10% polyacrylamide gel containing 0.1% gelatin (Sigma, St. Louis, USA). After electrophoresis, gels were soaked in 2.5% Triton X-100 for 45 minutes with single change of detergent solution. Gels were incubated for 18 h at 37°C in substrate buffer (50 mM Tris-HCl, pH 7.5, 5 mM CaCl_2_, and 0.02% NaN_3_), stained with 0.05% Coomassie brilliant blue G-250 (Sigma, St. Louis, USA), and destained in 10% acetic acid and 20% methanol. Gels were photographed and then quantitatedively measured by scanning densitometry. Each experiment was repeated three times.

### ELISA for VEGF

786O and ACHN cells were plated in 6-well tissue culture plates at a density of 1×10^6^ cells per well. Then, cells were transfected with pFlag-control and pFlag-RUNX3 with serum starvation. The supernatants were collected 24 h after transfection. VEGF concentration was determined using Quantikine ELISA kits according to the manufacturer's instructions (R&D Systems, MN, USA).

### Statistical analysis

Data are expressed as the means ± SD. Two-factor analysis of variance procedures and the Dunnett's t-test were used to assess differences within treatment groups. For TMA, statistical analysis was performed with SPSS 20 software (SPSS, Inc, Chicago, IL). The association between RUNX3 staining and the clinicopathologic parameters of the RCC patients, including age, gender, tumor size, grade, pT status and TNM stage, was evaluated by two sided Fisher's exact tests. Differences were considered significant when *P*<0.05.

## Results

### RUNX3 expression is decreased in human RCC

**Figure 1:**
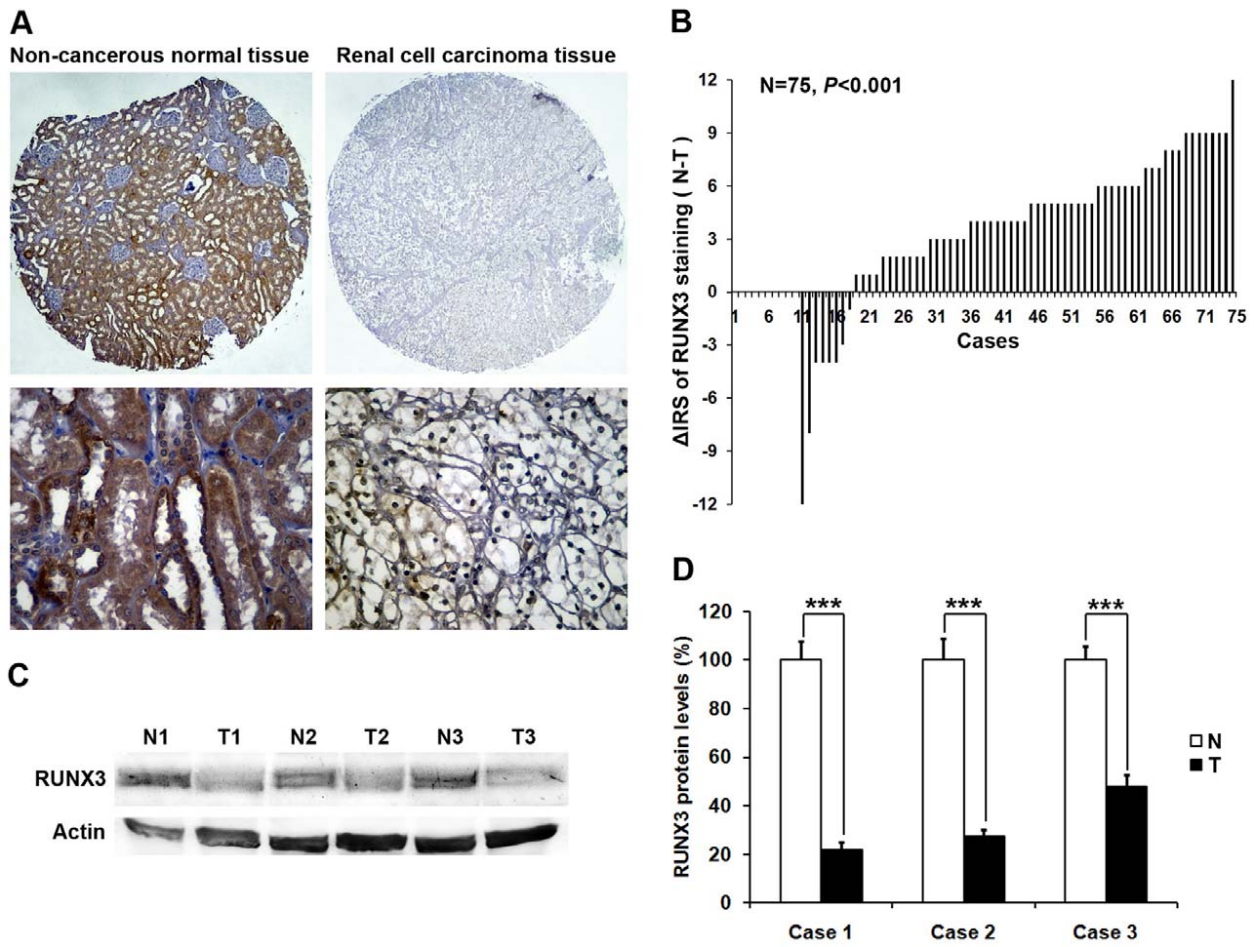
RUNX3 protein expression in RCC tissues and paired non-cancerous tissues. A Representative photographs showed RUNX3 immunohistochemical staining in TMA, which were taken at different magnifications (Top panel ×100, bottom panel ×400). B The distribution of the difference in RUNX3 staining (ΔIRS = IRS_N_-IRS_T_). Immunoreactivity score (IRS) of RUNX3 staining was available from 75 pairs of tissues; P values were calculated with the Wilcoxon test. C Whole-cell protein extracts were further prepared from three paired tumor adjacent normal renal tissues (N) and RCC tissues (T). The RUNX3 protein level was determined by Western blot analysis. Data are shown as mean ± SD. ***P<0.001.

We first determined whether RUNX3 expression is changed in human RCC. Immunohistochemistry staining was performed in TMA slide containing RCC tissues and paired non-cancerous tissues. The representative pictures presented in Fig. 1A showed that RUNX3 protein in nucleus was stained in brown. A significantly lower expression of RUNX3 was observed in the carcinoma tissues (*P* = 0.000, Fig. 1B). To further confirm these observations, Western blot assay was done using three RCC tissues and paired non-cancerous tissues. It was clear that the cancerous tissue had a drastic decrease of RUNX3 expression as compared with the non-cancerous tissues (Fig. 1C and D), which was consistent with the level of RUNX3 protein expression determined by immunohistochemical staining. These results showed that RUNX3 was commonly expressed in normal human renal cells but decreased or absent in renal cancer cells.

### Correlation of RUNX3 expression with clinicopathological parameters

**Figure 2:**
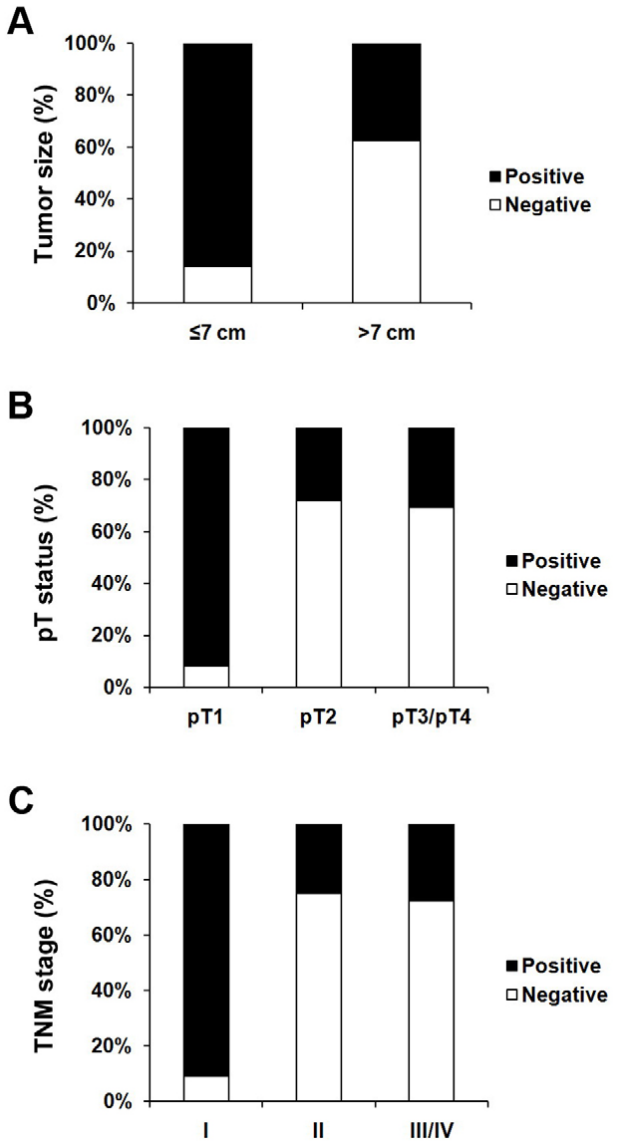
Correlation between RUNX3 expression and clinicopathologic parameters in RCC. A Decreased RUNX3 expression was correlated with tumor size (P<0.001, χ2 test, comparing ≤7 cm versus >7 cm). B Decreased RUNX3 expression was correlated with depth of invasion (P<0.001, χ2 test, comparing pT1 versus pT2–pT4). C Decreased RUNX3 expression was correlated with TNM stage (P<0.001, χ2 test, comparing I versus II–IV).

The clinicopathologic features of the 75 RCC biopsies were summarized in Table 1. There were 50 men and 25 women. Their average (SD) age of patients was 58.7 years (median, 56 years; range, 29–82 years). Our data showed that decreased expression of RUNX3 showed a significant correlation with tumor size (*P*<0.001, Fig. 2A). We also found decreased RUNX3 expression was significantly correlated with depth of invasion (comparing pT1 versus pT2–pT4) (*P*<0.001, Fig. 2B). Because TNM stage is an important prognostic marker for patients with RCC, we studied if RUNX3 expression correlates with TNM stage. We found RUNX3 staining was dramatically decreased in TNM stages II–IV compared with stage I (*P*<0.001, Fig. 2C). However, we did not find significant correlation between RUNX3 expression with other clinicopathologic variables, including age, gender and tumor grade.

**Table 1:** RUNX3 staining and clinicopathological characteristics of 75 renal cancer patients.

Variables	RUNX3 staining			
	Negative (%)	Positive (%)	Total	*P* *
**All cases Age**	26 (34.7)	49 (65.3)	75	
≤56 years	15 (41.7)	21 (58.3)	36	0.237
>56 years	11 (28.2)	28 (71.8)	39	
**Gender**				
Male	19 (38.0)	31 (62.0)	50	0.449
Female	7 (28.0)	18 (72.0)	25	
**Tumor size**				
≤7 cm	6 (14.0)	37 (86.0)	43	<0.001
>7 cm	20 (62.5)	12 (37.5)	32	
**Grade**				
I	0 (0.00)	3 (100.0)	3	0.132
II	9 (25.0)	27 (75.0)	36	
III	13 (46.4)	15 (53.6)	28	
IV	4 (50.0)	4 (50.0)	8	
**pT status**				
pT_1_	3 (8.1)	34 (91.9)	37	<0.001
pT_2_	18 (72.0)	7 (28.0)	25	
pT_3_	8 (66.7)	4 (33.3)	12	
pT_4_	1 (100.0)	0 (0.00)	1	
**TNM stage**				
I	3 (9.1)	30 (90.9)	33	<0.001
II	18 (75.0)	6 (25.0)	24	
III	12 (70.6)	5 (29.4)	17	
IV	1 (100.0)	0 (0.00)	1	

* Two sided Fisher's exact tests.

### Restoration of RUNX3 expression inhibits RCC cells migration and invasion in vitro

**Figure 3:**
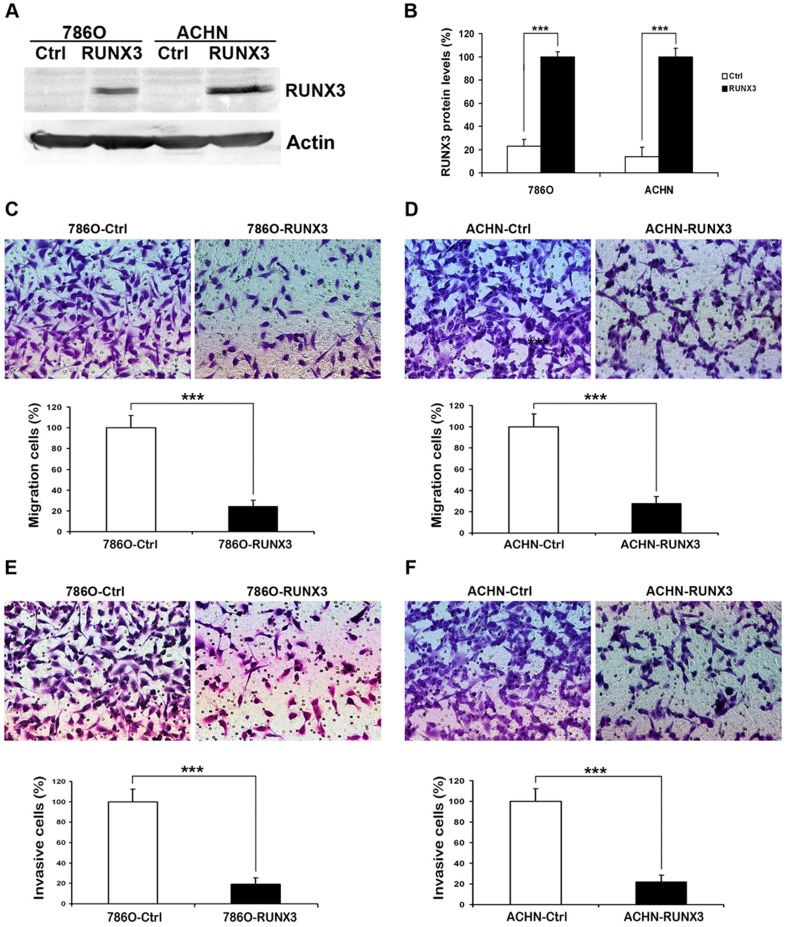
RUNX3 re-expression inhibits cell migration and invasion in 786O and ACHN cell lines. A Twenty-four hours after transfection, the expression of RUNX3 in 786O and ACHN cells was evaluated by Western blot. Actin was used as an internal control. B Quantitative analysis of Western blot shows that RUNX3 protein was over-expressed in 786O and ACHN after transfection. C, D Cell migration assay was performed after reintroduction of RUNX3 in 786O and ACHN cells. E, F Matrigel cell invasion assay was performed after re-expression of RUNX3 in 786O and ACHN cells. The data are presented as mean ± SD for triplicate determinations. *** P<0.001.

To determine the effect of RUNX3 reintroduction on RCC cells migration and invasion, we transiently transfected 786O and ACHN cells with pFlag-control and pFlag-RUNX3 plasmids. Twenty-four hours after transfection, RUNX3 protein was significantly over-expressed in cancer cells (Fig. 3A and B). Transfected cells were subjected to cell migration assay and invasion assay. In cell migration assay, we found that RUNX3 restoration in 786O and ACHN cells suppressed the ability to migrate through Boyden chamber by 76% and 72%, respectively (Fig. 3C and D). In cell invasion assay, RUNX3 restoration inhibited cell invasive ability of 786O and ACHN cells in matrigel-coated Boyden chamber by 81% and 78%, respectively (Fig. 3E and F). However, restoration of RUNX3 had no effect on the proliferation of RCC cells (Data not shown).

### Overexpression of RUNX3 reduces angiogenesis in vitro

**Figure 4:**
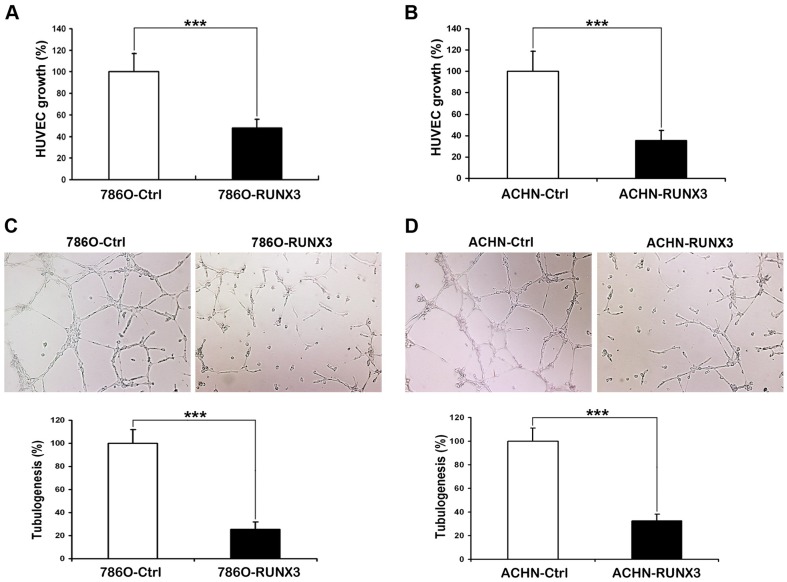
Reduction of angiogenesis in 786O and ACHN cells by restoration of RUNX3 expression. A, B CCK-8 cell proliferation assay was performed to detect the HUVECs proliferation. C, D Representative pictures were taken in situ for tube formation in the supernatant of 786O and ACHN cells. The degree of tube formation was assessed as the percentage of cell surface area versus total surface area. Control cell cultures were given arbitrary percentage values of 100. All experiments were carried out in triplicate. Data are shown as mean ± SD. ***P<0.001.

To further determine the effect of restored RUNX3 expression on angiogenic potential of human RCC cells, the angiogenic potentials of the supernatant of 786O and ACHN cells transfected with pFlag-control or pFlag-RUNX3 were determined by endothelial cell proliferation assay and tube formation assay. In the cell proliferation assay, we found that conditioned medium from 786O and ACHN cells transfected with pFlag-RUNX3 inhibited proliferation of endothelial cells compared with those of control cells (Fig. 4A and B). In the endothelial cell tube formation assay, the degree of tube formation was assessed as the percentage of cell surface area versus total surface area. As shown in Figure 4C and 4D, the average number of complete tubular structures formed by HUVECs was significantly reduced in conditioned medium from RUNX3-overexpressing cancer cells compared with vector controls.

### RUNX3 suppresses MMP-9 and VEGF expression and activity in RCC cells

**Figure 5:**
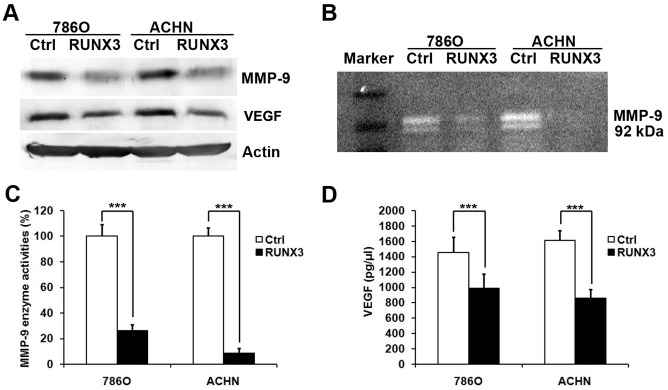
RUNX3 suppresses the expression of MMP-9 and VEGF in RCC cells. A Western blot analysis of the relative protein levels of MMP-9, VEGF and Actin in RUNX3 restoration and control group for both 786O and ACHN cell lines. B, C Gelatin zymography analysis of the enzyme activity of MMP-9 in RUNX3 re-expression and control group for both 786O and ACHN cell lines. D The secretion of VEGF was determined by ELISA assay. All experiments were carried out in triplicate. Data are shown as mean ± SD. ***P<0.001.

To investigate the mechanisms of RUNX3 regulating invasion and angiogenesis, we performed western blot, gelatin zymography and ELISA to detect the MMPs and VEGF levels in RCC cells. Our data showed that the MMP-9 and VEGF protein level was dramatically reduced in 786O and ACHN cells transfected with pFlag-RUNX3 (Figure 5A). The MMP-9 enzyme activity was significantly suppressed after RUNX3 expression in 786O and ACHN cells (Fig. 5B and C). And a significant inhibition in VEGF secretion was observed in conditioned medium from 786O and ACHN cells after transfection of pFlag-RUNX3 (Fig. 5D).

## Discussion

RUNX3 was originally cloned as AML2 and is localized on chromosome 1p36.1, which is among the most frequently affected regions in various types of cancers [13]. RUNX3 has multiple functions and was at first reported to correlate with the genesis and progression of human gastric cancer as a tumor suppressor. RUNX3-null mice exhibit hyperplasia of gastric mucosa as a result of stimulated proliferation and suppressed apoptosis of epithelial cells [14]. Besides gastric cancer, it has been reported that reduced expression of RUNX3 was observed in various kinds of cancers, including breast cancer, colorectal cancer, glioma and melanoma [9], [12], [15], [16]. It has been reported that reduced expression of RUNX3 was frequently caused by CpG island hypermethylation [17]. Moreover, point mutations of RUNX3 were observed in certain type of human cancers including gastric and bladder cancers [14], [17]. These observations suggested a tumor suppressor role for RUNX3 in human cancers. In the present study, we used TMA technology, immunohistochemistry and Western blot to investigate the role of RUNX3 in RCC. Our results demonstrated that RUNX3 expression was decreased in RCC tissues compared with tumor adjacent normal renal tissues (Fig. 1). Furthermore, we evaluated the association between RUNX3 expression and clinicopathological characteristics. Our data showed that decreased RUNX3 expression was drastically associated with tumor size, depth of invasion and TNM stage (Fig. 2). This implied that RUNX3 may play an important role in the RCC development and progression. However, the mechanism of RUNX3 expression in RCC still remains to be determined.

Transforming growth factor-β (TGF-β) signaling pathway is an essential regulator of cellular proliferation, invasion and migration in a variety of different cancer types [18], [19]. Malfunctions of TGF-β signaling are implicated in serious human cancers [20]. RUNX3 is a target of TGF-β-mediated tumor suppressor pathway. RUNX3 and other signal transducers such as Smad are collectively required for the tumor suppressor activity of the TGF-β pathway [7]. Given the potential role of RUNX3 in TGF-β signaling, it is possible that the tumor suppressor activity of RUNX3 is realized by regulating cell migration and invasion. In our study, we found that there were significant inhibitions of invasion as well as migration by re-expression of RUNX3 in RCC cell lines (Fig. 3).

Matrix metalloproteinases (MMPs) are a family of zinc-dependent endopeptidase that are capable of degrading components of the basement membrane and ECM, allowing cancer cells to migrate and invade [21], [22]. TGF-β pathway has been documented to contribute to signal transduction pathways in the regulation of MMP-2, MMP-9 and MMP-11 [23], [24]. In our previous study, we found that RUNX3, being a downstream molecule of TGF-β, inhibited glioma cell invasion and migration by regulating the MMP-2 protein expression and enzyme activity [12]. To determine how RUNX3 inhibits RCC cell migration and invasion, we focused on elucidating the relationship between RUNX3 and MMPs which has been reported to participate closely in tumor progression [25]. Here, we found that RUNX3 over-expression significantly inhibited the expression and bioactivities of MMP-9 in 786O and ACHN cells (Fig. 5A–C). Our data indicate that RUNX3 may suppress RCC cell invasion and migration through decreasing MMP-9 protein expression and inhibiting enzyme activity. However, it remains to be elucidated how RUNX3 regulates MMP-9 expression and activity and its signal pathway to regulate RCC cell invasion.

In addition to the invasion and migration changes brought out by RUNX3, it appears to exert its tumor suppressor activity through antiangiogenic [11]. In the present study, we found that the transfection of pFlag-RUNX3 reduced the capacity of RCC cells supernatant to stimulate proliferation and tube formation of human endothelial cells compared with those of control cells, suggesting that restoration of RUNX3 expression significantly impaired angiogenic potential of RCC cells in vitro (Fig. 4).

Of the numerous angiogenic factors discovered thus far, VEGF has been identified as a key mediator of tumor angiogenesis involved in the development of tumor blood supply in the progression of solid tumors [26]. Peng demonstrated that RUNX3 down-regulated VEGF expression via transcriptional repression in human gastric cancer. We detected the expression and secretion of VEGF after RUNX3 transfection. Our data showed that VEGF expression and secretion was decreased by restoration of RUNX3 (Fig. 5A, D). These results suggested that RUNX3 suppresses blood vessel formation by regulating VEGF secretion.

In general, this study provides evidence that RUNX3 was expressed at low levels in RCC. Decreased RUNX3 expression is significantly with RCC progression. The lost of RUNX3 expression may contribute to the tumor invasion and growth. Re-expression of RUNX3 leads to the inhibition of RCC cell migration and invasion by decreasing MMP-9 protein expression and suppressing enzyme activity. We further demonstrated that restoration of RUNX3 reduced proliferation and blood vessel formation of HUVECs through decreasing the expression and secretion of VEGF. These results suggested that targeting of the RUNX3 pathway may constitute a potential treatment modality for RCC.
